# Advances in galactose-based small-molecule hepatocyte-targeting probes for detecting biomarkers in hepatocellular carcinoma

**DOI:** 10.3389/fchem.2026.1899981

**Published:** 2026-08-20

**Authors:** Xin Li, Jiajia Fu, Meixia Wang, Zhiwei Zhu

**Affiliations:** 1 Department of Oncology, Jingshan People’s Hospital, Jingshan, China; 2 Guangxi Key Laboratory of Special Biomedicine, School of Medicine, Guangxi University, Nanning, China; 3 Department of Pediatrics, Jingshan People’s Hospital, Jingshan, China; 4 Department of Pediatrics, Hunan Provincial People’s Hospital, First Affiliated Hospital of Hunan Normal University, Changsha, Hunan, China

**Keywords:** diagnosis, fluorescent probe, galactose, hepatocellular carcinoma, hepatocyte-targeting

## Abstract

Hepatocellular carcinoma (HCC) is a type of malignant tumor with a low early detection rate, high morbidity, and high mortality. Therefore, early diagnosis of HCC is essential for improving patient survival and treatment outcomes. Traditional diagnostic approaches generally exhibit insufficient specificity and sensitivity. However, small-molecule fluorescent probes offer advantages, such as non-invasiveness, high sensitivity, and real-time imaging capabilities, are considered ideal tools for liver cancer research, and are widely used in imaging and treatment studies. Asialoglycoprotein receptor (ASGPR) is overexpressed on hepatoma cells, and galactose can be recognized by ASGPR via the cluster glycoside effect; hence, various galactose-based small-molecule hepatocyte-targeting fluorescent probes have been developed. These probes can target hepatocytes via receptor-mediated endocytosis and release a fluorescent dye upon reactions with specific small molecules or enzymatic biomarkers, making them powerful tools for the early diagnosis of HCC. The present work is a review of recent advances in galactose-based small-molecule hepatocyte-targeting probes for detecting HCC-related markers and provides a detailed account of their physicochemical properties and applications in cellular and biological systems. We also conduct an in-depth examination of existing issues and provide a summary of the future trends and challenges. We hope that this review may promote the development of novel fluorescent probes for HCC detection and further provide meaningful guidance for the diagnosis and treatment of HCC.

## 1 Introduction

**SCHEME 1:**
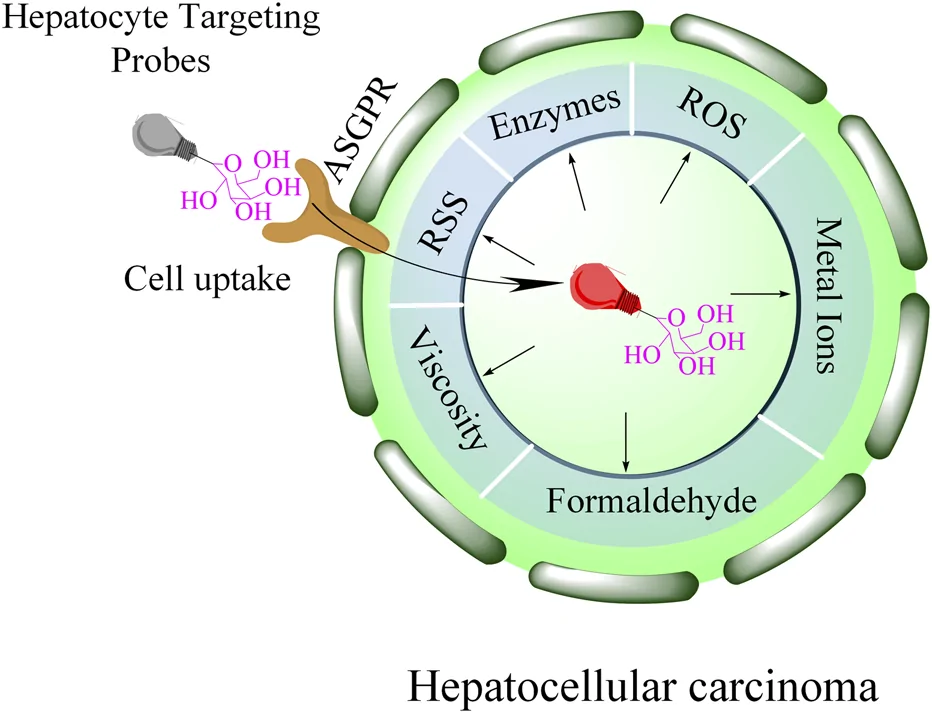
Schematic illustration of galactose-based small-molecule hepatocyte-targeting probes and their bioapplications.

The liver is a major organ involved in glycogen and lipid storage and protein synthesis that plays a crucial role in human metabolic processes (Luo et al., 2024; Trefts et al., 2017). However, despite its formidable capabilities, the liver is susceptible to various diseases, such as HCC, which pose a grave threat to human health (Miao et al., 2021; Ohashi et al., 2018). HCC is a highly invasive malignant tumor and is the most common type of primary liver tumor; it accounts for over 80% of all primary liver cancer cases and ranks as the third leading cause of cancer mortality worldwide (HoChong et al., 2022; Mazzanti et al., 2008; Quan et al., 2023). Consequently, exploring novel diagnostic strategies tailored to the characteristics of HCC is of great importance. The traditional methods used for detecting and diagnosing HCC include magnetic resonance imaging, computed tomography, positron-emission tomography, and ultrasound (Bonanni et al., 2014; Chiu et al., 2024; Pinkoviezky et al., 2025; Renzulli and Giampalma, 2025). However, these techniques exhibit limitations in HCC imaging, such as low resolution, lengthy imaging time, limited specificity and sensitivity, and high costs (Ding et al., 2022; Jin et al., 2018; Li et al., 2020; Liu et al., 2025). Small-molecule fluorescence probe imaging technology is characterized by high sensitivity, good visualization, non-invasiveness, and real-time detection and has become crucial in the early diagnosis of HCC (Jiang et al., 2020; Jin et al., 2002; Liu et al., 2018; Liu et al., 2022; Guo et al., 2014). Research has indicated that galactose can be specifically recognized by the ASGPR overexpressed on hepatocytes (Bernardes et al., 2008; Linghu et al., 2024; Wang et al., 2022a). Moreover, galactose-based small-molecule hepatocyte-targeting fluorescent probes have become a prominent research topic for imaging HCC in recent years (Liao et al., 2025; Liu et al., 2023; Zhou et al., 2021). Although various galactose-based small-molecule hepatocyte-targeting probes have been developed to date, a comprehensive review of their applications in the detection of HCC remains relatively underexplored. The present review primarily examines recent advances in galactose-based small-molecule hepatocyte-targeting fluorescent probes for HCC fluorescence imaging. These probes have been widely applied to detect various HCC-related biomarkers (Scheme 1), including enzymes (β-galactosidase/β-Gal), reactive oxygen species (ROS; e.g., hypochlorite/HClO), reactive sulfur species (RSS; e.g., cysteine/Cys and H_2_S), metal ions (Fe^3+^ and Hg^2+^), endogenous formaldehyde (FA), and microenvironmental factors (e.g., viscosity).

### 2 Synthesis and conjugation strategies for galactose-based small-molecule hepatocyte-targeting fluorescent probes

The development of galactose-based fluorescent probes depends on the availability of efficient synthesis strategies. These strategies require effective coupling of the galactose ligands to fluorophores while maintaining both ASGPR-targeting ability and the optical properties of the fluorophores. This review primarily discusses two synthesis approaches (Table 1): O-glycosidic bond formation and click-chemistry-mediated coupling. Typically, fully acetyl-protected bromo-galactose is used as the glycosyl donor, which undergoes a nucleophilic substitution reaction with the phenolic hydroxyl group on the fluorescent moiety to form an O-glycosidic bond, yielding an acetyl-protected glycoside intermediate. This intermediate is then deacetylated to yield a probe modified with galactose (Chen et al., 2021). The advantage of this strategy is its well-established synthetic route and the dual roles of galactose as both a targeting agent and a responsive moiety (Chen et al., 2019). Click chemistry has become an efficient tool for constructing galactose probes; this strategy involves the reaction of galactosyl azide with a terminal-alkyne-functionalized fluorophore under CuSO_4_/sodium ascorbate catalysis to form a stable 1,2,3-triazole bond (Lai et al., 2013), which offers advantages such as mild reaction conditions, excellent regional selectivity, and flexible modular design (Zhao et al., 2023).

**TABLE 1:** Summary of the synthesis and conjugation strategies of representative probes.

Probe name	Linkage chemistry	Galactose valency	Synthesis advantages	Principal limitations	References
DCM-Mor-Gal	O-glycosidic bond	Monovalent	O-glycosidic bond with dual targeting and response functions	Copper catalyst required (cytotoxicity risk)	Yi et al. (2025)
LDMTY	O-glycosidic bond	Monovalent	High yield of the target compound (62% yield)	Dual-lock limits applicability in tissues lacking either of the two biomarkers	Liao et al. (2025)
TCF-Gal-Cys	1,2,3-Triazole (click chemistry)	Monovalent	High-efficiency linkages via click chemistry	Galactose is used solely for targeting (no responsive function)	Linghu et al. (2024)
DCM-Cys-Gal	1,2,3-Triazole (click chemistry)	Monovalent	High-efficiency linkages via click chemistry	Galactose is used solely for targeting (no responsive function)	Li et al. (2024b)
QL-Gal-N_3_	1,2,3-Triazole (click chemistry)	Monovalent	Excellent water solubility	The yield of the final product is extremely low (19%)	Shen et al. (2020)
MOR-HOCl-Gal	1,2,3-Triazole (click chemistry)	Monovalent	Classical rhodamine spirocycle synthesis (acid-catalyzed, well-established)	Lengthy reaction time (step a: 24 h; step b: 12 h)	Qin et al. (2024)
TP-Gal-1	1,2,3-Triazole (click chemistry)	Monovalent	Well-established naphthalimide platform (mild synthesis, easy derivatization)	Multistep synthesis; purification difficulty increases with higher valency	Liu et al. (2023)
TP-Gal-2	Same as TP-Gal-1	Divalent	Same as TP-Gal-1	Same as TP-Gal-1	Liu et al. (2023)
TP-Gal-3	Same as TP-Gal-1	Trivalent	Same as TP-Gal-1	Same as TP-Gal-1	Liu et al. (2023)
Rho-Gal	1,2,3-Triazole (click chemistry)	Monovalent	Classic rhodamine B scaffold (facile synthesis, good photophysical properties)	Strict deprotection pH (9–10); over-alkalinity may hydrolyze spirocycle	Hu and Wang (2022)
Rho-Lac	_	_	_	_	Hu and Wang (2022)
Rho-Glu	_	_	_	_	Hu and Wang (2022)
NFP-G	O-alkyl glycosidic bond +1,2,3-triazole (click chemistry)	Monovalent	Stable alkyl glycosidic ether linkage	The yield in the final step is extremely low (16%)	Zhou et al. (2021)
HT-V	O-glycosidic bond	Monovalent	Short synthesis (four steps)	Inherent hydrophobicity; difficult to modify	Li et al. (2022)

## 3 Galactose-based small-molecule hepatocyte-targeting fluorescent probes for detecting HCC-associated enzymes

Enzymes are regarded as critical substances for the early diagnosis of cancer (Gu et al., 2019; Hu et al., 2019; Sakabe et al., 2012). Multiple types of cancers exhibit overexpression of specific enzymes, making real-time monitoring of enzyme activity *in vivo* crucial for early tumor diagnosis (Baig et al., 2019; Mooney et al., 2017). Many fluorophores, such as rhodamine, oxazinone, naphthylamide, and dansylamide, have been exploited to construct fluorescent probes for enzymatic detection (Gao et al., 2022; Hu et al., 2024; Li et al., 2024a; Liu et al., 2025). However, there are few reports of specific fluorescent probes for detecting enzymes associated with HCC, especially enzyme-responsive hepatocyte-specific small-molecule fluorescent probes. In HCC, β-Gal activity is markedly upregulated in tumor tissues and has been validated as a clinically valuable biomarker for rapid tumor identification (Ogawa et al., 2021). The development of enzyme-responsive hepatocyte-specific small-molecule fluorescent probes is therefore crucial and urgently needed for the early detection and treatment of HCC.

**FIGURE 1:**
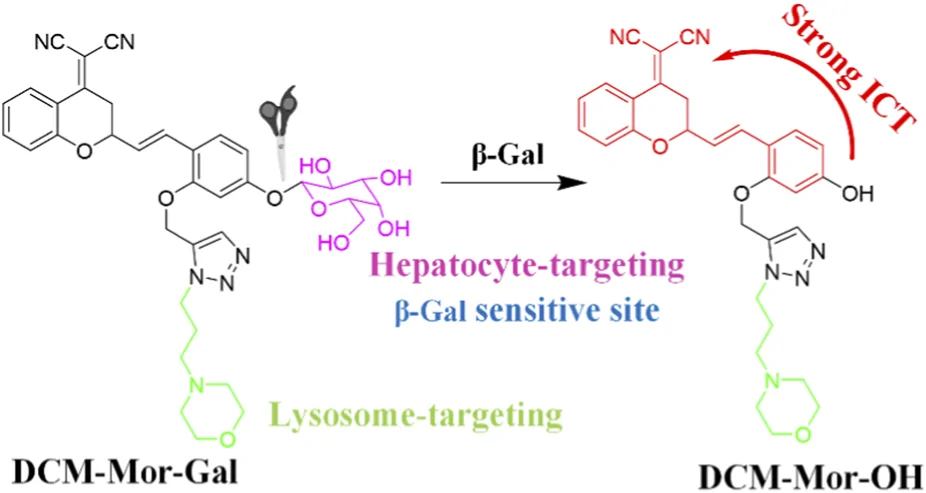
Recognition mechanism of the DCM-Mor-Gal probe for β-Gal.

Recently, Yi et al. (2025) developed a new near-infrared (NIR) fluorescent probe called DCM-Mor-Gal (Figure 1), which can sensitively detect β-Gal activity and target liver cancer cells *in vivo*; in this work, galactose was selected not as the liver-cell-targeting moiety but as the responsive group for β-Gal. The glycosidic bond formed between galactose and the fluorescent group of the probe served as the recognition site for β-Gal. The response mechanism for the DCM-Mor-Gal probe was as follows: the glycosidic bond of DCM-Mor-Gal was cleaved by β-Gal to produce DCM-Mor-OH and promote intramolecular charge transfer (ICT) of DCM-Mor-Gal, thereby enhancing its fluorescence. The probe possessed the advantages of NIR emission and a large Stokes shift (130 nm), which aided in the detection of biological tissue samples. The fluorescence intensity of DCM-Mor-Gal reached its maximum within 15 min and remained stable for 30 min, indicating its great significance for experimental observation and data collection. Furthermore, DCM-Mor-Gal exhibited excellent light stability and maintained outstanding analytical performance in the pH range of 6.0–10.0. In addition, the limit of detection (LOD) of β-Gal was 7.6 × 10^−3^ U/mL, and the addition of increasing amounts of β-Gal to the DCM-Mor-Gal probe solution produced a clear fluorescence emission peak at 690 nm. This peak gradually increased with the increase of β-Gal, and the peak intensity was positively correlated with β-Gal concentration, exhibiting good sensitivity for DCM-Mor-Gal toward β-Gal. More importantly, DCM-Mor-Gal could distinguish hepatocytes (HepG2) from SGC7901, HeLa, A549, and human normal hepatocytes (HL-7702) effectively via galactosyl group mediation; it could also aggregate effectively within lysosomes under the influence of acid-base attraction, demonstrating significant dual targeting potential for hepatocytes and lysosomes. Zebrafish experiments reported by the authors verified the efficacy of DCM-Mor-Gal in detecting β-Gal activity *in vivo*. In conclusion, the DCM-Mor-Gal probe offers novel avenues for clinical diagnosis of liver-related diseases and is expected to be an effective tool for investigating their pathological mechanisms.

**FIGURE 2:**
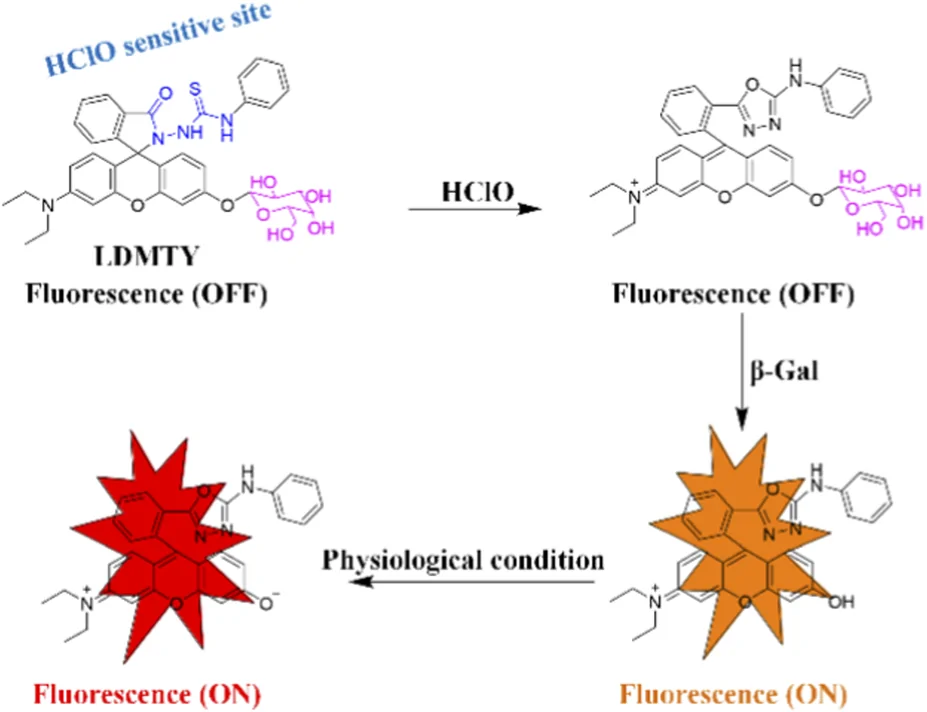
Recognition mechanism of the LDMTY probe for β-Gal and HClO.

More recently, Liao et al. (2025) designed and synthesized a novel fluorescent probe called LDMTY (Figure 2), which was the first tandem dual-locked fluorescent probe that could identify the simultaneous overexpressions of β-Gal and HClO in HCC while possessing liver-targeting capabilities. Compared to the DCM-Mor-Gal probe, LDMTY offers enhanced specificity for HCC through a tandem dual-lock design. LDMTY utilized the fluorescent dye rhodamine as the fluorophore, which possesses low cytotoxicity and high fluorescence efficiency, along with a thiocyanate ester that could rapidly undergo cyclization with HClO as the HClO-responsive switch; in addition, a galactose unit was conjugated to the structure through an ether bond, forming a glycosidic bond that served as an additional switch for the probe. As with DCM-Mor-Gal, the galactose unit served as the hepatocyte-targeting group. These two switches collectively formed the tandem dual-locked function. Notably, the presence of β-Gal or HClO alone did not elicit a substantial fluorescence response. LDMTY rapidly responded to β-Gal detection only when both stimuli were present, maintaining its dual-locked state in the absence of either target. LDMTY displayed high sensitivity and specificity to β-Gal and HClO with minimum LODs of 2.54 × 10^−4^ U/mL and 0.046 μM, respectively. Upon addition of β-Gal and HClO to the LDMTY solution, the fluorescence intensity increased rapidly and reached its peak value within the first 30 min. Moreover, the fluorescence remained stable under continuous illumination at 520 nm for 0–60 min, demonstrating its rapid response and excellent photostability. In the presence of β-Gal and HClO, LDMTY exhibited the highest fluorescence intensity under neutral conditions (pH = 7.4) and retained its strong fluorescence in the slightly acidic tumor microenvironment, indicating its potential for tumor detection applications. Furthermore, LDMTY could distinguish cancer cells from normal cells based on discrepancies in the intracellular basal β-Gal and HClO levels, particularly in HCC cells that exhibited prominent fluorescent signal characteristics. LDMTY was also found to be capable of effectively monitoring the dynamic changes in endogenous HClO present within living organisms and has been successfully applied to *in vivo* imaging studies in mouse HCC models. LDMTY is an innovative tandem dual-locked visual analysis tool with high specificity, high sensitivity, and outstanding liver-targeting capacity; it demonstrates considerable promise and is expected to provide significant value for the specific detection of HCC.

## 4 Galactose-based small-molecule hepatocyte-targeting fluorescent probes for detecting RSS

The liver is one of the main organs responsible for the synthesis of RSS, such as Cys and H_2_S. A recent fluorescent probe study confirmed that Cys levels are significantly elevated in HCC cells compared to normal hepatocytes (Jin et al., 2026). H_2_S production and signaling in the liver are altered in HCC, and H_2_S is also recognized as a liver-function-related biomarker. A two-photon fluorescent probe has been successfully applied to distinguish clinical HCC tissues from adjacent normal tissues (Liu et al., 2024). RSS play irreplaceable roles in physiological and pathological processes (Giles and Jacob, 2002; Bilska-Wilkosz and Iciek, 2022; Checa et al., 2020); overexpression of RSS can contribute to the development of associated diseases, including inflammatory disorders, cancer, and Alzheimer’s disease, and is frequently observed in various malignancies such as liver cancer (Reja et al., 2017; Zhang et al., 2020). Many methods of RSS detection have been reported, such as high-performance liquid chromatography, mass spectrometry, electrochemical detection, spectrophotometry, gas chromatography, and fluorescence imaging (Chen et al., 2023; Dong et al., 2025; Giedroc et al., 2023; Zeng et al., 2021; Zhang et al., 2026; Zhou et al., 2022). However, these approaches are not designed for detecting RSS in hepatocytes specifically.

**FIGURE 3:**
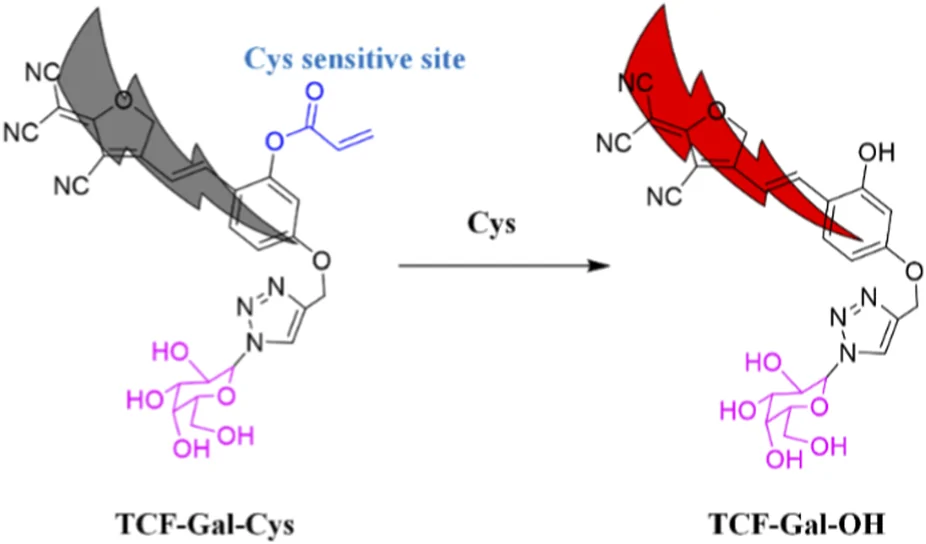
Recognition mechanism of the TCF-Gal-Cys probe for Cys.

Linghu et al. (2024) created a NIR hepatocyte-targeting fluorescence probe called TCF-Gal-Cys (Figure 3) to monitor Cys-related diseases *in vitro* and *in vivo.* The TCF-Gal-Cys probe comprised tricyanofuran (TCF) as the fluorophore, galactose as the recognition moiety for ASGPRs on hepatocytes, and an acrylate group as the Cys response site. The TCF-Gal-Cys probe offers excellent visualization capability; upon adding Cys to a solution of TCF-Gal-Cys, the solution distinctly changed from light yellow to light pink. In addition, the fluorescence intensity of the probe solution was linearly correlated with Cys concentration, with the peak at 645 nm increasing as the concentration of Cys increased from 0 to 100 μM; the LOD of TCF-Gal-Cys was as low as 0.08 μМ, displaying a highly sensitive fluorescence turn-on response to Cys. The TCF-Gal-Cys solution incubated with Cys did not emit obvious fluorescence in an acidic environment but displayed a significant fluorescence response to Cys in the pH range of 6–11, indicating that TCF-Gal-Cys could be used to detect Cys in biological systems stably. The fluorescence intensity of the probe solution was unaffected by other amino acids, representative cations and anions, glutathione (GSH), and homocysteine (Hcy), showing excellent selectivity toward Cys. No obvious changes in fluorescence intensity were observed upon adding GSH and Hcy to the probe solution, which demonstrated that the probe could distinguish between Cys and other thiols. Moreover, TCF-Gal-Cys showed low cytotoxicity, high security, good membrane permeability, and potential to detect endogenous Cys levels in liver cells. Because the terminal galactose structure of TCF-Gal-Cys could be internalized by HepG2 cells overexpressing ASGPRs, TCF-Gal-Cys could specifically distinguish hepatocellular cells from A549, HeLa, and SGC-7901 cells while exhibiting excellent hepatocyte-targeting capability. In addition, the TCF-Gal-Cys probe demonstrated exceptional imaging performance for Cys in zebrafish, as the NIR fluorescence emitted by TCF-Gal-Cys was able to penetrate zebrafish tissues and eliminate interference caused by autofluorescence within the organism. The liver-targeting TCF-Gal-Cys probe could thus serve as an excellent imaging tool for investigating Cys-related physiological and pathological issues and can be used for real-time monitoring of intracellular and *in vivo* Cys levels.

**FIGURE 4:**
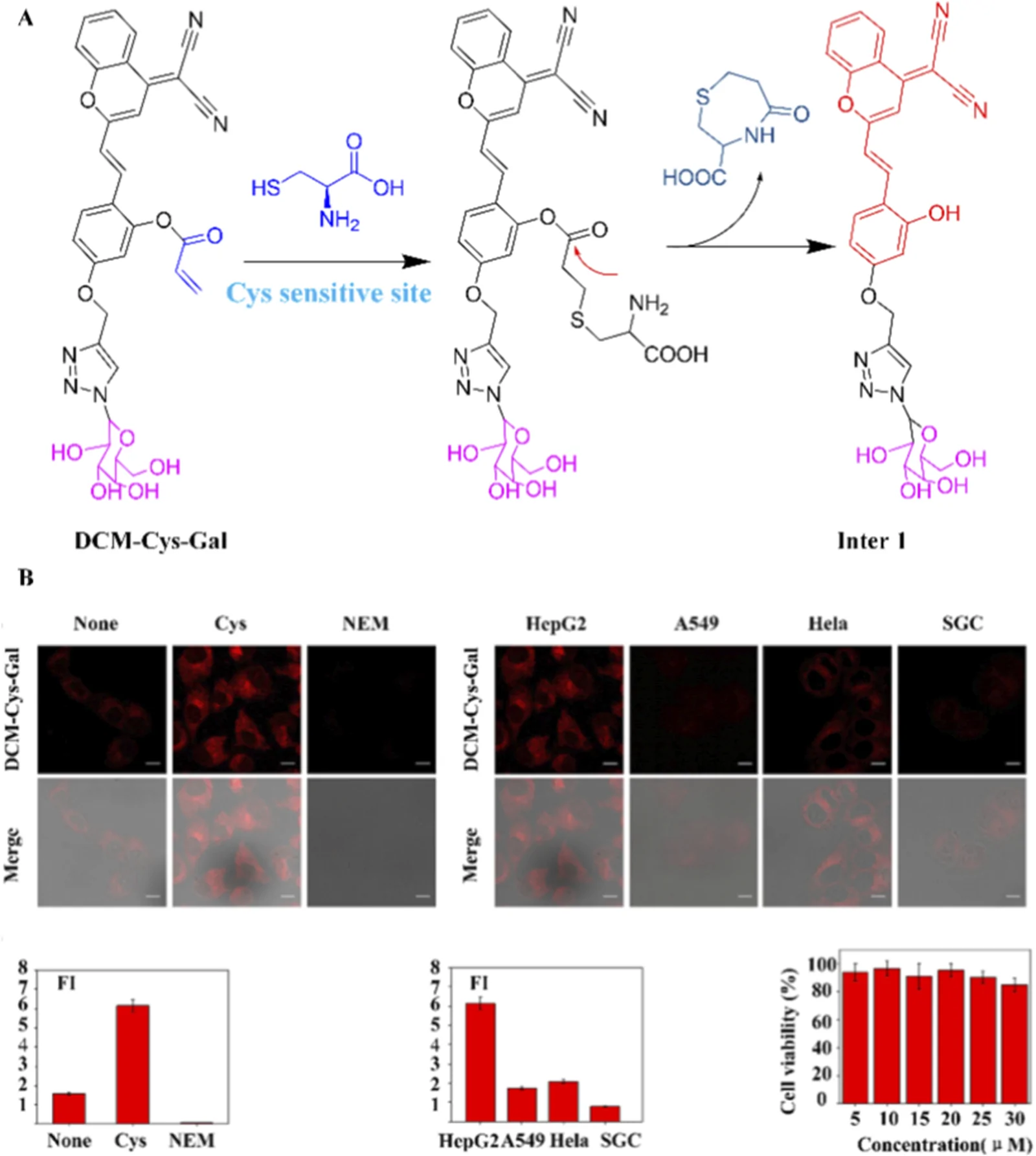
**(A)** Recognition mechanism of the DCM-Cys-Gal probe for Cys. **(B)** Visual detection of Cys at the cellular level using DCM-Cys-Gal and its hepatocyte-targeting capacity. Reprinted from Li et al. (2024b) with permission from Elsevier, Copyright 2024.

In 2024, researchers developed another hepatocyte-targeting fluorescent probe called DCM-Cys-Gal (Figure 4A) for detecting Cys in living cells and zebrafish (Li et al., 2024b). The probe comprised acrylate as the Cys-responsive moiety and galactose as the hepatocyte-targeting group, with dicyanomethylene-4H-pyran (DCM) serving as the fluorophore owing to its long-wavelength emission and high quantum yield. Similar to the TCF-Gal-Cys probe, the DCM-Cys-Gal probe underwent intramolecular cyclization following reaction with Cys to form a free seven-membered ring and release the fluorophore Inter 1. The DCM-Cys-Gal probe possessed excellent visualization capability similar to that of TCF-Gal-Cys, and the probe solution changed from yellow to pink upon reaction with Cys, indicating that DCM-Cys-Gal can be used to visually detect Cys. The reaction rate between DCM-Cys-Gal and Cys was rapid such that the fluorescence intensity of the probe solution increased obviously once Cys was added, reaching the maximum intensity within 20 min and remaining stable for 50 min. No significant changes were observed in the fluorescence intensity of the DCM-Cys-Gal solution in the physiological pH range. However, upon addition of Cys, the fluorescence intensity increased significantly at 723 nm and decreased gradually at 668 nm, demonstrating that the probe was stable under physiological pH conditions and could be used for Cys detection in biological systems. The DCM-Cys-Gal probe exhibited relatively high sensitivity to Cys and could be used to detect Cys both qualitatively and quantitatively *in vitro* with a low LOD of 7.7 × 10^−7^ M and a fluorescence quantum yield of 0.185. In addition, DCM-Cys-Gal displayed superior selectivity toward Cys; the fluorescence of the DCM-Cys-Gal solution was remarkably enhanced upon adding Cys and remained unaffected by other biothiols, anions, and cations, including tryptophan, leucine, Cl^−^, ClO^−^, Na^+^, and Mg^2+^. Moreover, DCM-Cys-Gal exhibited low cytotoxicity and could be used to effectively differentiate hepatocytes from A549, HeLa, and SGC-7901 cells, demonstrating excellent hepatocyte-targeting properties (Figure 4B). More importantly, DCM-Cys-Gal possessed the characteristics of low light scattering and high penetration depth, which were used to successfully visualize fluorescence imaging in a zebrafish model; this indicated that DCM-Cys-Gal had significant potential in detecting Cys *in vivo.* Therefore, this hepatocyte-targeting fluorescent probe may be useful for characterizing liver cancer cells and studying Cys-related liver diseases.

The DCM-Cys-Gal probe displayed a longer NIR emission wavelength, while the TCF-Gal-Cys probe performed better in terms of response time and LOD. TCF-Gal-Cys showed direct evidence of galactose-mediated targeting through fluorescence imaging experiments on HepG2 cells treated with galactose, whereas DCM-Cys-Gal-targeting of hepatocytes was inferred indirectly from its cell selectivity. These findings highlight the importance of integrating specific considerations in future designs of probes to maximize their diagnostic potential for HCC.

**FIGURE 5:**
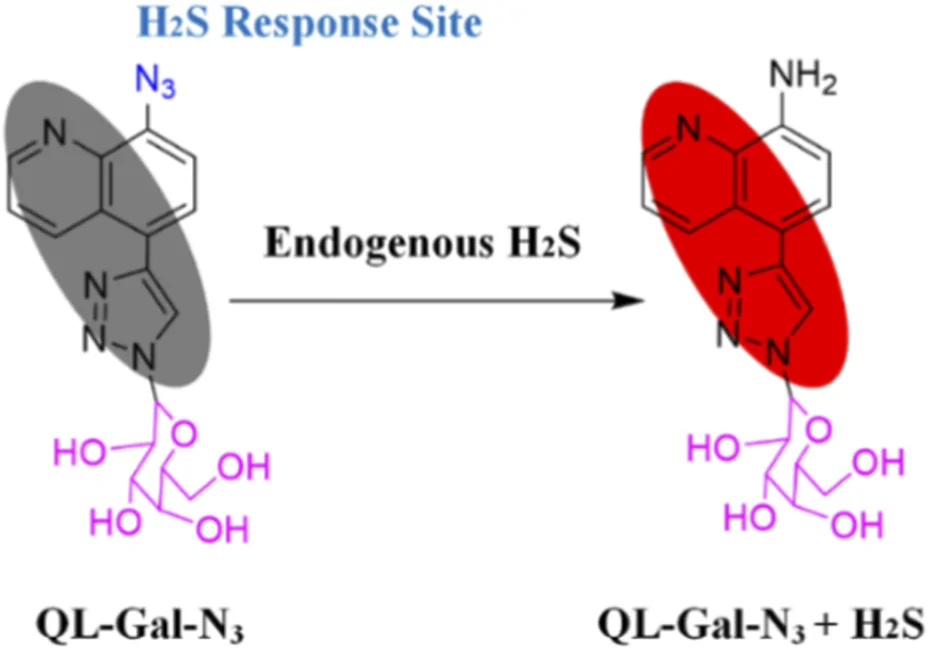
Recognition mechanism of the QL-Gal-N_3_ probe for H_2_S.

Shen et al. (2020) designed and synthesized a novel fluorescent probe called QL-Gal-N_3_ for detecting H_2_S based on an H_2_S-mediated azide reduction strategy (Figure 5). Here, the azide group of the probe molecule was reduced to an amino group by H_2_S, which eliminated the photoinduced electron transfer (PET) effect of the fluorophore and allowed it to regain its original fluorescence emission capacity, resulting in significant enhancement of the fluorescence. The QL-Gal-N_3_ probe exhibited excellent selectivity toward H_2_S. Upon addition of H_2_S, the probe solution showed marked fluorescence enhancement at 521 nm, with the intensity increasing by nearly 102-fold. In contrast, no obvious fluorescence response was observed with Cys, GSH, or Hcy, and these species showed little effect on the detection process. The QL-Gal-N_3_ probe displayed great pH stability over the pH range of 4–10 and achieved maximum fluorescence intensity at pH 7, indicating that the probe was suitable for detecting H_2_S under physiological conditions. However, in an acidic environment, the fluorescence emission intensity was significantly lower, indicating that thiolysis of the azido bond was impaired in strongly acidic solutions. In addition, the QL-Gal-N_3_ probe responded rapidly to H_2_S at 521 nm under physiological conditions, with the fluorescence intensity increasing quickly and reaching a plateau within approximately 1 min. The fluorescence emission intensity demonstrated excellent linearity with H_2_S concentration from 0 to 100 μM; the LOD of the probe to H_2_S was 1.26 × 10^−7^ M, which suggested that QL-Gal-N_3_ had a notably low detection limit and was appropriate for detecting H_2_S in water samples. More importantly, QL-Gal-N3 exhibited low cytotoxicity and was successfully applied to detect H_2_S in hepatocytes. It also displayed higher selectivity toward HepG2 cells than A549 and HeLa cells, showing good application prospects for accurate H_2_S detection in hepatocytes.

## 5 Galactose-based small-molecule hepatocyte-targeting fluorescent probes for detecting ROS

ROS encompass various oxygen anions, peroxides, and oxygen-containing free radicals, all of which are the byproducts of aerobic metabolism in cellular organisms for regulating various physiological and biological responses as intermediaries in intracellular signaling (Mittal et al., 2014; Reczek and Chandel, 2015). Various conditions, including inflammation, liver damage, coronary atherosclerotic heart disease, and cancer, are related to the abnormal accumulation of ROS (Kattoor et al., 2017; Ochoa et al., 2018; Prieto and Monsalve, 2017; Wang et al., 2021b; Moloney and Cotter, 2018; Wang et al., 2021a). Recently, ROS-activated fluorescent probe imaging technology has advanced rapidly and has great clinical significance (Shi et al., 2026; Wang et al., 2026a; Zhou et al., 2026). The development of ROS-activated hepatocyte-specific small-molecule fluorescent probes for early diagnosis of HCC is both crucial and urgent yet remains limited. Hence, we provide an in-depth review of relevant studies.

**FIGURE 6:**
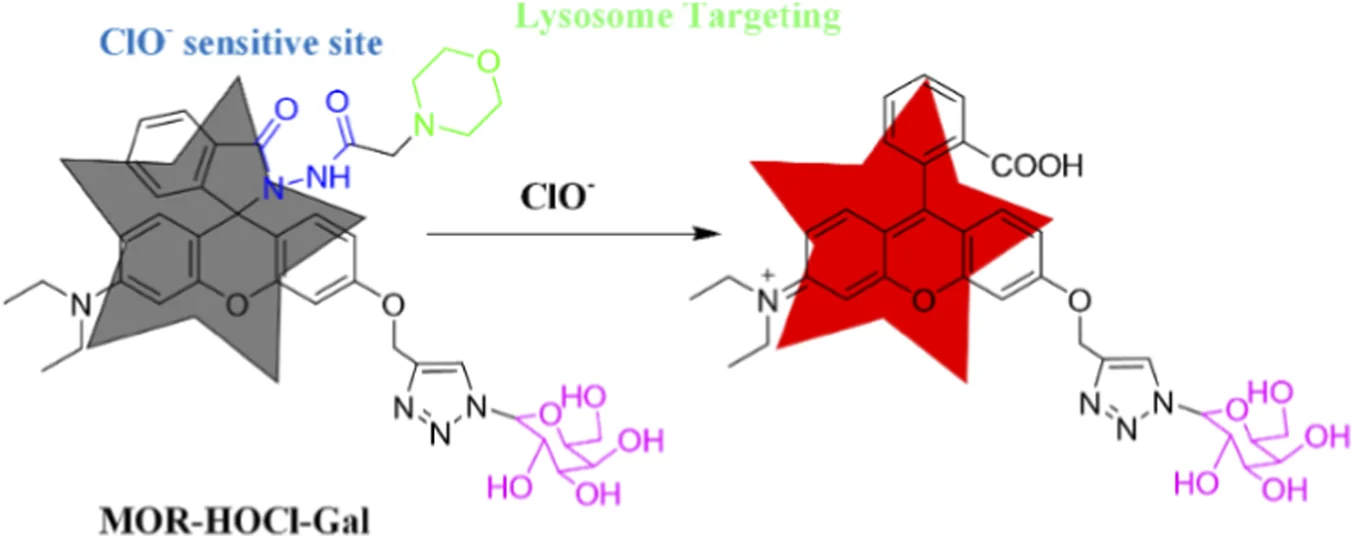
Recognition mechanism of the MOR-HOCl-Gal probe for ClO^−^.

HClO has been identified as a tumor-associated biomarker because its levels are significantly elevated in HCC cells compared to normal hepatocytes (Li et al., 2023). To achieve early diagnosis of HCC, Qin et al. (2024) designed and synthesized a novel hepatocyte-targeting and lysosomal-targeting probe called MOR-HOCl-Gal (Figure 6). This probe incorporated a hydrazide group as the ClO^−^ recognition receptor owing to its outstanding specificity, *β*-galactose as the hepatocyte-targeting unit, morpholine as the lysosome-targeting moiety, and a rhodamine derivative with favorable photophysical properties as the fluorophore. MOR-HOCl-Gal could be used for both qualitative and quantitative detection of ClO^−^ *in vitro* with significant sensitivity. The probe solution exhibited strong fluorescence at 560 nm upon adding ClO^−^, and the fluorescence intensity increased with increasing ClO^−^ concentration. The LOD of MOR-HOCl-Gal toward ClO^−^ was 1.4 × 10^−7^ M. The MOR-HOCl-Gal probe displayed good selectivity toward ClO^−^ as the fluorescence of the probe solution was significantly enhanced upon the addition of ClO^−^ without interference from other ROS, representative anions, and cations. In addition, MOR-HOCl-Gal exhibited relatively low toxicity and could be used to monitor endogenous or exogenous ClO^−^ in cells, indicating its potential for safe detection in biological systems. The MOR-HOCl-Gal probe could distinguish hepatoma cells from A549, MCF7, and LoVo cells while specifically reaching the intracellular lysosomes of living cells; this ability was successfully applied to visualize ClO^−^ in live zebrafish. Thus, MOR-HOCl-Gal is expected to become a novel and highly efficient diagnostic tool for monitoring ClO^−^ levels in liver-related diseases precisely.

## 6 Galactose-based small-molecule hepatocyte-targeting fluorescent probes for detecting metal ions

**FIGURE 7:**
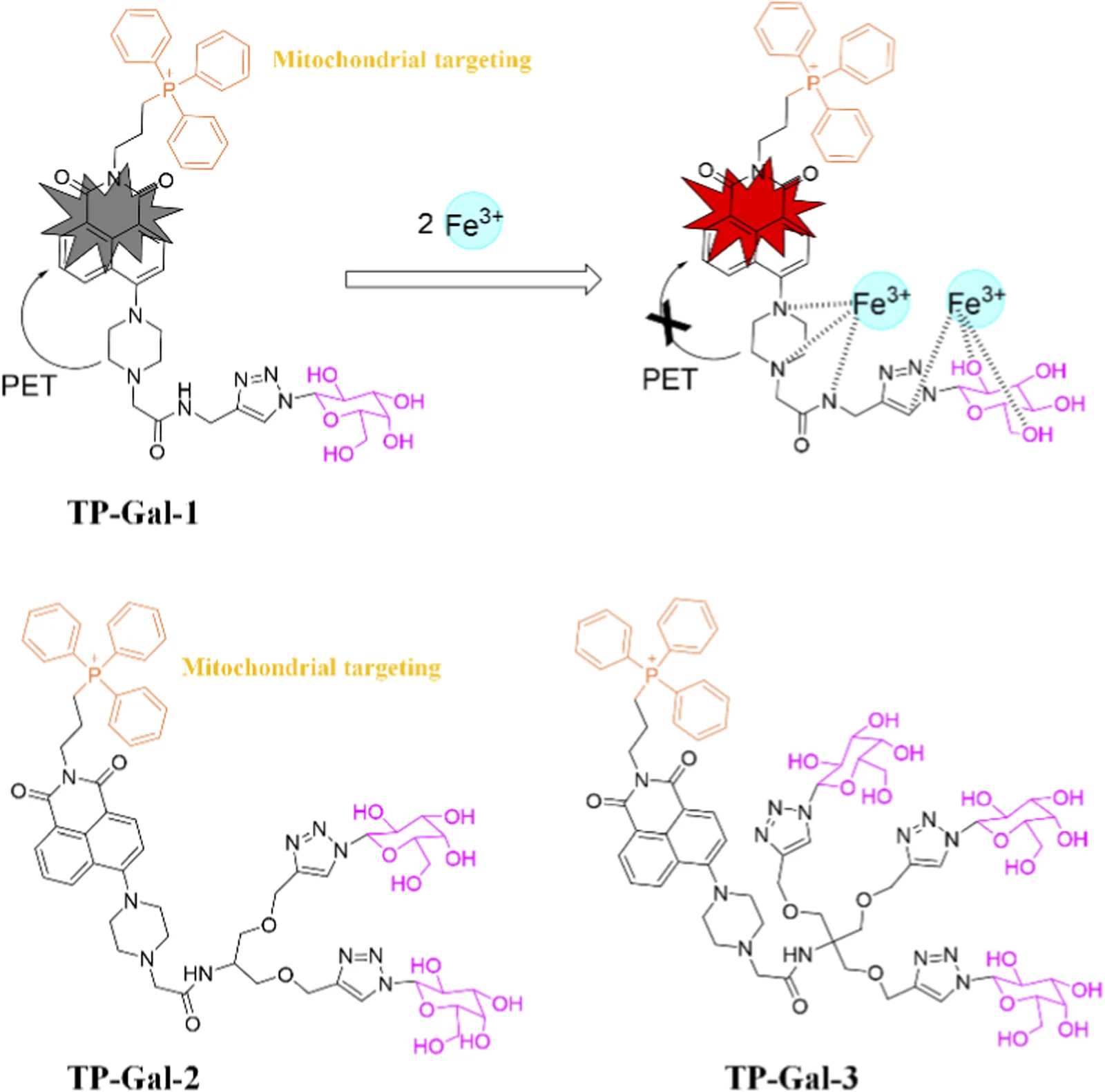
Structures of TP-Gal-1, TP-Gal-2, and TP-Gal-3, along with the recognition mechanism of the TP-Gal-1 probe for Fe^3+^.

Several heavy-metal ions, Fe^3+^, and Hg^2+^ can also be used as biomarkers to detect HCC. Earlier research suggests that abnormal levels of Fe^3+^ or Hg^2+^ could lead to conditions, such as neurodegenerative disorders, liver damage, and even cancers (Duan et al., 2018; Ke et al., 2018; Li et al., 2021; Liu et al., 2017; Wang et al., 2022b). Iron overload has been recognized as a sensitive diagnostic and prognostic biomarker because iron metabolism is dysregulated in HCC and contributes to disease progression (Chatzikalil et al., 2025). To resolve such issues, three novel dual-cascade-targeting (hepatocyte and mitochondria) fluorescent probes (i.e., TP-Gal-1, TP-Gal-2, and TP-Gal-3) based on a glycocluster strategy were reported by Liu et al. (2023) to detect Fe^3+^ in hepatic mitochondria; the authors used TP-Gal-1 as an example to explain its response mechanism to Fe^3+^ (Figure 7). The binding of Fe^3+^ to the piperazine nitrogen atom of TP-Gal-1 inhibited PET from piperazine to 1,8-naphthylimide, causing TP-Gal-1 to adopt a “fluorescence-on” state and emit intense fluorescence. The three probes (TP-Gal-1, TP-Gal-2, and TP-Gal-3) showed considerable selectivity toward Fe^3+^ as their fluorescence intensities increased significantly at 530 nm upon addition of Fe^3+^, along with markedly enhanced fluorescence quantum yields. Moreover, the probes exhibited exceptional sensitivity to Fe^3+^, with all three probes demonstrating prominent concentration-dependent increases in fluorescence intensity at 530 nm in the presence of Fe^3+^ and displaying relatively obvious fluorescence enhancements in response to Fe^3+^ in the physiological pH range. The LODs of the probes toward Fe^3+^ were as follows: 7.90 × 10^−8^ M for TP-Gal-1, 5.06 × 10^−8^ M for TP-Gal-2, and 1.04 × 10^−7^ M for TP-Gal-3. These probes could serve as potential candidates for Fe^3+^ detection in solution given their favorable selectivity and sensitivity. Owing to the presence of the “sugar cluster effect,” the liver-targeting capability exhibited the following trend: TP-Gal-1 (monogalactosyl) < TP-Gal-2 (digalactosyl) < TP-Gal-3 (trigalactosyl); here, TP-Gal-3 exhibited the most favorable liver-targeting capability among the three probes. The TP-Gal series of probes directly demonstrated galactose-mediated targeting through the “sugar cluster effect,” whereby the liver-targeting efficiency showed an upward trend as the number of galactose units increased, which was confirmed both at the cellular level and in organ distribution in mice. Moreover, with the assistance of the positively charged triphenylphosphine, the probes demonstrated excellent mitochondrial localization capabilities and successfully detected Fe^3+^ in the mitochondria of HepG2 cells but not in HeLa, A549, or SGC7901 cells lacking ASGPRs. Overall, these bispecifically targeted galactosylated probes were highly promising detection tools for identifying Fe^3+^ within hepatocyte mitochondria; this holds significant importance for the precise diagnosis and in-depth understanding of liver mitochondrial Fe^3+^-related diseases.

**FIGURE 8:**
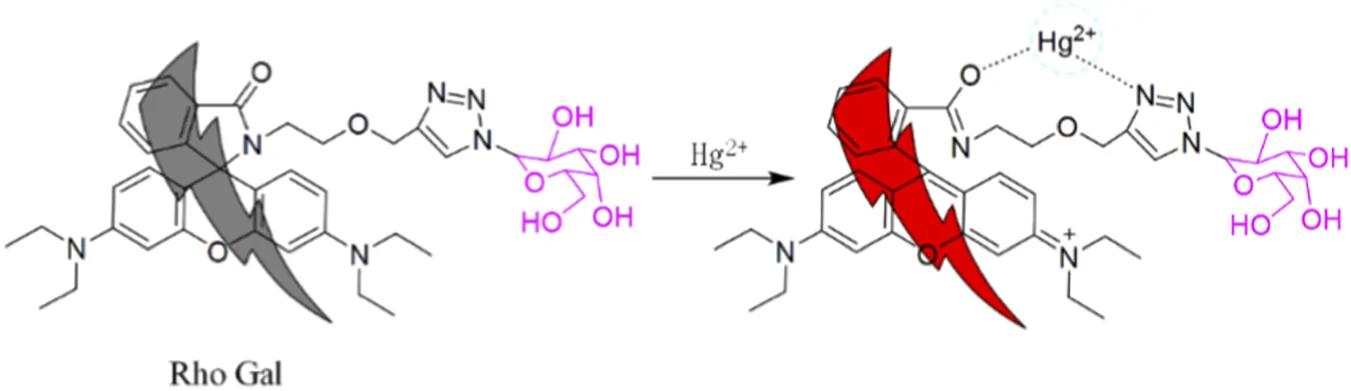
Recognition mechanism of the Rho-Gal probe for Hg^2+^.

Studies have shown that Hg^2+^ exposure alters the expressions of more than 2,200 genes in the HepG2 cells. These include genes involved in cell-cycle regulation, apoptosis, and DNA repair, all of which are closely associated with the onset and progression of liver cancer (Ayensu and Tchounwou, 2006). Hu and Wang (2022) developed three liver-targeting fluorescent probes (Rho-Gal, Rho-Lac, and Rho-Glu) for detecting Hg^2+^ in hepatocytes (Figure 8). These probes were capable of specifically recognizing Hg^2+^ in an aqueous solution. Upon adding Hg^2+^, the fluorescence activities of Rho-Gal, Rho-Lac, and Rho-Glu were enhanced greatly, with the strongest signal being observed at 582 nm. In contrast, no obvious fluorescence enhancement was observed upon the addition of other ions. The probe solution of Rho-Gal with Hg^2+^ ions changed to a red color after ultraviolet irradiation at 365 nm, demonstrating excellent visualization capability. The fluorescence responses of the probe–Hg^2+^ complexes were strong in the pH range of 3.5–6.5 with weak interference from the pH value, indicating that the probes were suitable for fluorescence imaging of Hg^2+^ in a slightly acidic microenvironment. These probes also exhibited good sensitivity toward Hg^2+^, and the fluorescence intensity improved with increasing Hg^2+^ concentration, revealing good concentration dependence. The LODs of the probes toward Hg^2+^ were as follows: 9.49 × 10^−8^ M for Rho-Gal, 1.37 × 10^−8^ M for Rho-Lac, and 1.33 × 10^−8^ M for Rho-Glu. These characteristics demonstrated that the probes exhibited outstanding physicochemical properties and could thus be used to qualitatively and quantitatively detect Hg^2+^ in the solution. In addition, these three probes were capable of responding to Hg^2+^ in a cellular environment. However, the probes were barely recognized by dead HepG2, HeLa, and SGC7901 cells lacking ASGPRs. In contrast, a significant difference was observed in the fluorescence response to Hg^2+^ in HepG2 cells, with the response following the trend Rho-Gal > Rho-Lac > Rho-Glu; this is because Rho-Gal carries a galactose moiety, enabling it to be recognized by live HepG2 cells overexpressing ASGPRs and to selectively enter the cells via receptor–ligand interactions. Rho-Gal was non-cytotoxic and labeled the HepG2 cells selectively. Therefore, it could be safely used to detect the internalized Hg^2+^ in hepatocytes. However, none of the three probes have been validated through animal studies; thus, the *in vivo* targeting capabilities and metabolic behaviors remain unclear. Accordingly, *in vivo* pharmacokinetic and biodistribution studies are needed to validate the clinical translation potentials of these probes.

## 7 Galactose-based small-molecule hepatocyte-targeting fluorescent probes for detecting endogenous FA

Research demonstrates that the FA levels in healthy tissues or cells typically range from 100 to 400 μM (Heck and Casanova, 2004; Heck et al., 1982) but may reach as high as 1,000 μM in tumor cells (Hauptmann et al., 2009). Abnormally high levels of FA in organisms can trigger a variety of diseases, such as neurodegenerative disorders, tissue cancerization, and hepatitis (Brewer and Chang, 2015; Liu et al., 2019; Paudel et al., 2025). Long-term exposure to FA can disrupt the balance of lipid metabolism in liver cells, and lipid metabolic reprogramming is one of the hallmark features of HCC (Bai et al., 2017). Therefore, abnormal endogenous FA level may reflect a state of metabolic stress in HCC, making it a potentially valuable disease-related biomarker. Investigating the presence of FA in liver cancer cells is of great significance for understanding the relationship between endogenous FA and liver disease.

**FIGURE 9:**
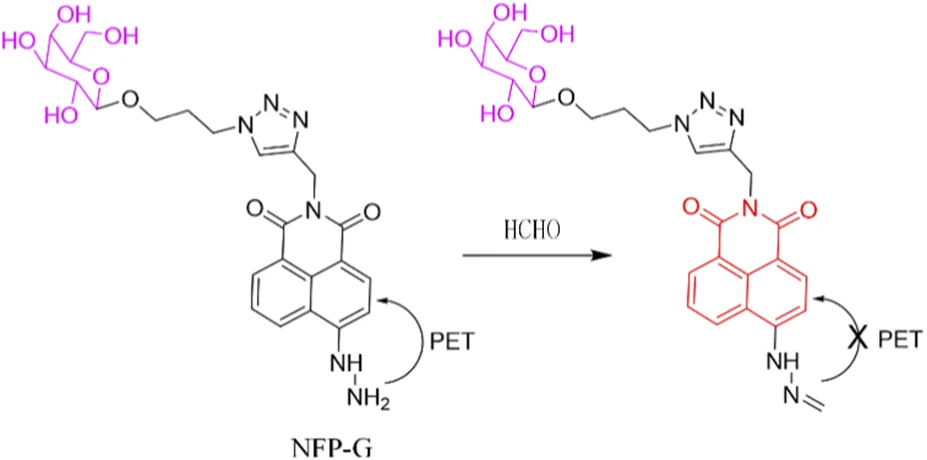
Recognition mechanism of the NFP-G probe for formaldehyde.

Zhou et al. (2021) introduced a galactose-guided fluorescent probe called NFP-G for selectively bioimaging endogenous FA in living HepG2 cells (Figure 9); here, the NFP-G probe utilized *β*-D-galactose as the targeting moiety, employed a hydrazine group as the receptor for FA, and used 1,8-naphthylimide as the fluorophore. The response mechanism between NFP-G and FA was such that the primary amine underwent a condensation reaction with FA to form hydrazine, thereby inhibiting the PET process and resulting in a marked increase in fluorescence. The NFP-G probe exhibited high selectivity toward FA. The fluorescence enhancements produced by interactions with sodium pyruvate, acetaldehyde, ethylene glycol, and propionaldehyde were significantly lower than that with FA, indicating that the probe had potential for FA detection in real samples. In addition, the NFP-G probe offered excellent stability; the fluorescence intensity at 541 nm remained almost unchanged in the pH range of 7–8. NFP-G also displayed great sensitivity, and a good linear relationship was observed between the fluorescence intensity and FA concentration, with the detection limit of NFP-G toward FA being 1.2 × 10^−7^ M. NFP-G also exhibited low cytotoxicity: the survival rates of L929, HeLa, and HepG2 cells were all above 90% following treatment with 10 μM of the probe. More importantly, the strong affinity between *β*-D-galactose and the ASGPR allowed NFP-G to specifically enter the HepG2 cells, enabling successful application of the probe for imaging FA in living HepG2 cells. To verify the liver-targeting ability of the probe, a unique strategy was employed: after pretreatment with β-Gal to remove the galactosyl group, the probe exhibited fluorescence in all cell lines and lost its selectivity toward liver cells. This “loss-of-function” experiment directly demonstrated that the galactose moiety was a necessary condition for liver targeting at the molecular level, and this evidence was more persuasive than indirect inferences based solely on comparisons of cell selectivity. This study may supply some novel insights into the design of fluorescent probes for detecting certain substances in tumor cells. However, NFP-G exhibited a relatively long response time (60 min), and no animal studies were conducted, which may limit its applications in rapid detection and *in vivo* imaging.

## 8 Galactose-based small-molecule hepatocyte-targeting fluorescent probes for detecting viscosity

**FIGURE 10:**
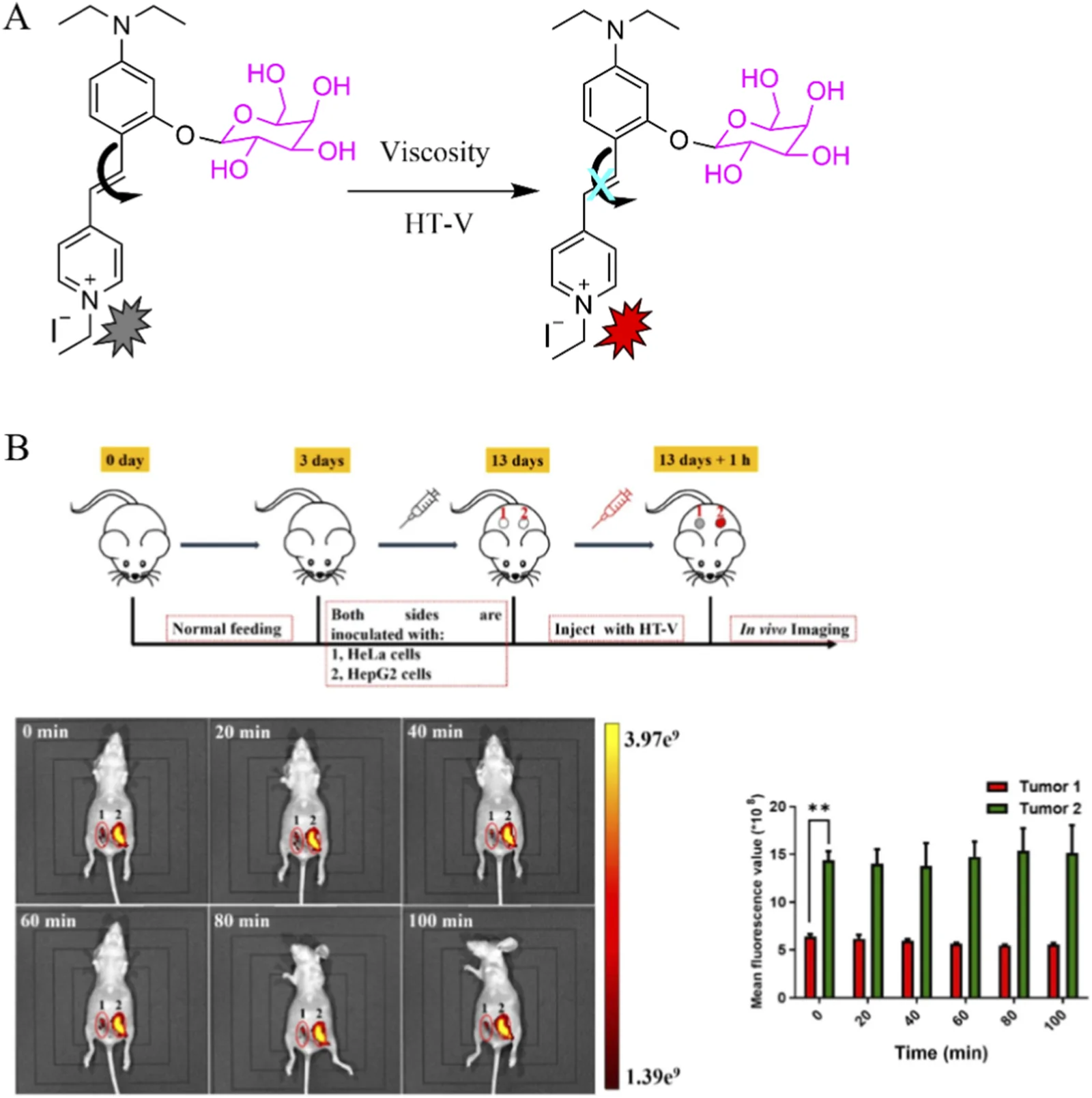
**(A)** Recognition mechanism of the HT-V probe for viscosity. **(B)** Liver tumor specificity imaging with the HT-V probe. Reprinted from Li et al. (2022) with permission from the American Chemical Society, Copyright 2022.

Viscosity is a very important indicator of the microenvironment in an organism and plays a crucial role in regulating the physical or chemical activities of certain molecules (Pei et al., 2026; Wang et al., 2026b; Yang et al., 2014). Many biological processes, including protein folding, viscosity-dependent catalysis, and signal transduction, are highly dependent on viscosity for maintaining equilibrium. Abnormal changes in viscosity can disrupt cellular functions and lead to the development of various diseases, such as Alzheimer’s disease, atherosclerosis, diabetes, and cancer (Bera et al., 2022; Li et al., 2025; Wang et al., 2023; Zhang et al., 2024a). Research has shown that the liver viscosity increases abnormally in the early stages of HCC and can serve as a specific imaging target for HCC (Luo et al., 2025; Zhang et al., 2024b). Therefore, investigating the liver viscosity level is highly significant for diagnosing and treating liver cancer. To address this issue, Li et al. (2022) engineered a hepatocyte-specific fluorescent probe called HT-V for imaging and detecting viscosity in HCC (Figure 10A); here, galactose was used as the hepatocyte-targeting moiety owing to its specific recognition by the ASGPR, and an ether bond was employed to link the galactose residue to the fluorophore. HT-V exhibited good viscosity detection with clear visualization *in vitro*. Under natural light, the color of HT-V was yellow in water but red in glycerol. The fluorescence intensity of HT-V displayed an excellent linear relationship with viscosity, which indicated that HT-V could be used to quantitatively determine viscosity. In addition, the HT-V probe exhibited good light stability and was highly sensitive to changes in viscosity in the pH range of 5–11, which ensured more accurate viscosity measurements. The HT-V probe was found to not react with other substances; the fluorescence intensity at 616 nm was significantly enhanced only in the presence of sufficient viscosity and remained essentially unchanged in different polar solvents, indicating great applicability in complex biological systems. Similar to the TCF-Gal-Cys probe, the addition of D-galactose to HepG2 cells resulted in a gradual decrease in intracellular fluorescence, demonstrating that the hepatocyte-targeting effect was mediated by the galactose moiety. Furthermore, HT-V exhibited low cytotoxicity and could be used for live cell imaging; it showed strong fluorescence in HepG2 cells but almost no fluorescence in other cancer cells, indicating selective targeting of hepatocytes. The probe could also be used for organ imaging; it showed a stronger fluorescence signal in HepG2 xenograft tumors than normal liver tissues, with the fluorescence intensity being approximately 3-fold higher. The probe displayed excellent photostability in tissues as its fluorescence intensity remained mostly unchanged for about 100 min. More importantly, the HT-V probe could be used to specifically image tumors within the liver; in a related study, HeLa and HepG2 cells were implanted subcutaneously into nude mice to establish tumor models (HeLa cells as tumor 1 and HepG2 cells as tumor 2), and HT-V was injected into these tumors. There was a considerable difference in fluorescence intensity between the two tumors, with the fluorescence intensity of tumor 1 being much weaker than that of tumor 2 (Figure 10B). This result demonstrated that galactose exhibited promising targeting capacity and specificity for HCC, indicating that the HT-V probe has significant potential for HCC detection and may be suitable for *in situ* imaging.

### 9 Summary and outlook

In this review, we comprehensively summarized recent advances in galactose-based hepatocyte-targeting probes and their applications for HCC detection and investigation (Tables 2–4). These probes have been found to possess excellent physical and chemical properties while enabling real-time, non-invasive, and visible fluorescence imaging in cells, tissues, or animal models, thereby providing a valuable complement to traditional methods. The liver-targeting fluorescent probes explored herein were ingeniously designed to target hepatocytes for the detection of relevant pathological biomarkers, including various enzymes (β-Gal), RSS (Cys, H_2_S), ROS (HOCl), metal ions (Fe^3+^, Hg^2+^), endogenous FA, and viscosity, thus providing powerful tools with immense potential for both biological research and the diagnosis of liver cancer. Despite the remarkable achievements of fluorescence imaging technology in detecting biomarkers associated with HCC, the path to realizing future applications of fluorescent probes in clinical diagnosis and treatment remains long. To advance the future development of fluorescent probes aimed at detecting HCC-related biomarkers, the following factors are recommended.

1. Expanding the types of target sites for the probes: The majority of galactose-based small-molecule hepatocyte-targeting probes reported to date were designed to target only a single analyte (such as those responding only to β-Gal or Cys). In the complex tumor microenvironment, signaling activation driven by a single biomarker is susceptible to background expression in normal tissues or non-specific interference, which can lead to the generation of false-positive signals. In recent years, design strategies for dual-response or multiresponse probes have gradually attracted attention. For example, the LDMTY probe detects β-Gal and HClO simultaneously, which are closely associated with HCC; a “double-lock” mechanism has been implemented here that activates the fluorescent response only when both conditions are met simultaneously, effectively improving the specificity of the diagnosis. However, multiresponse probes also face challenges, such as complex molecular design, difficult synthesis, potential cross-reactions between different responsive groups, and a more cumbersome validation process. Therefore, there is a need to design optimized probes tailored to specific scenarios to achieve precise detection of HCC.
2. Advancing the development of NIR region II (NIR-II, 1,000–1,700 nm) galactose-based small-molecule hepatocyte-targeting probes: Compared to the NIR-I region, light scattering and autofluorescence interference are significantly lower in the NIR-II region, which enables a higher signal-to-noise ratio and deeper tissue penetration (up to several centimeters). This holds tremendous potential for intraoperative navigation of liver tumors and precise localization of deep-seated lesions. However, small-molecule probes that target the liver and emit in the NIR-II wavelength range are still very scarce at present. Hence, it is crucial to develop novel NIR-II fluorescent dyes and rationally conjugate them with galactose-targeting groups to construct an NIR-II probe system with high quantum yield, good biocompatibility, and liver-specific targeting.
3. Currently, the applications of many probes are influenced by factors such as probe concentration and excitation light intensity. In the future, ratio-based liver-targeted fluorescent probes should be developed, which can eliminate external interference using a built-in reference signal to enable quantitative imaging of biomarkers and provide data support for the early diagnosis of HCC.
4. In addition to β-Gal, Cys, HClO, and metal ions, which have been studied extensively, there are many other biomarkers closely associated with the onset and progression of HCC that have not been fully developed as probe targets. Thus, it is important to explore promising new biomarkers for HCC. (a) γ-Glutamyltransferase (GGT) has been found to be persistently elevated in the sera of HCC patients, and its activity levels are significantly correlated with tumor burden and poor patient prognosis. Furthermore, the active site of GGT is well-defined, and there are precedents for the successful application of GGT-responsive probes in other cancers (Hu et al., 2024), providing a solid foundation for their adaptation to HCC probes. (b) Aminopeptidase N (APN/CD13) is a membrane-bound enzyme that is highly expressed on the cell membrane of HCC cells; it is directly involved in tumor invasion, migration, and angiogenesis. Its catalytic domain is located on the extracellular side of the cell, facilitating direct recognition by probes without the need for intracellular entry, making it an ideal membrane-surface target (Li et al., 2020). (c) Leucine aminopeptidase (LAP), whose activity reflects the extent of hepatocyte damage, is known to be significantly upregulated in HCC tissues and is closely associated with postoperative recurrence of HCC (He et al., 2017). Incorporating these biomarkers into the design of targeted fluorescent probes for HCC is expected to expand the range of targets for HCC detection and improve the ability to diagnose HCC.
5. Strengthen clinical validation studies and rigorously establish the specificity of new biomarkers: To date, the majority of studies have been limited to cell lines, zebrafish, and mice models, and there is a lack of validation data based on clinical samples. Here, we suggest adopting the following multistep verification scheme. (a) Screening at the cellular level by comparing the HCC cell lines with non-liver cancer cell lines, such as HeLa, A549, and SGC7901, to verify probe selectivity for liver cancer cells. (b) Competitive inhibition experiments involving preincubating with specific enzyme inhibitors or natural substrates and observing the degree of attenuation in the fluorescence response of the probe to confirm the specificity of the signal source. (c) Collecting surgical resection specimens, biopsy tissues, and serum samples from patients with HCC and performing tissue microarray analysis to investigate the associations between the fluorescence signal of probe and pathological grade, clinical stage, and prognostic indicators of patients. (d) Performing immunohistochemical analyses on the same tissue sections or enzyme-linked immunosorbent assay (ELISA) evaluations on the same serum samples to cross-validate the probe signals against expression levels of the target biomarkers, thereby confirming specificity at the protein level. This multistep strategy would systematically bridge the gap between fundamental research and clinical translation.
6. Exploring the design of galactose-targeted probes for integrated diagnosis and treatment: To date, there have been very few reports on therapeutic galactose-targeted probes with HCC imaging and therapeutic capabilities. This field is still in its early stages of exploration. However, two recent studies have provided strong support for an integrated diagnosis and treatment strategy. Ai et al. (2025) coupled a NIR fluorophore to ursodeoxycholic acid via an ester bond to develop an esterase-activated fluorescent prodrug, enabling the early diagnosis and concurrent treatment of liver fibrosis. It is worth noting that a GalNAc-modified triple-lock photodynamic probe developed by Lu et al. (2025) enables active targeting of the liver through GalNAc–ASGPR interactions. This approach enables selective NIRF/PA dual-modality imaging and type I photodynamic therapy of senescent cells in a liver fibrosis model by leveraging a three-step response mechanism involving β-Gal, acidic pH, and high viscosity, providing direct evidence of the feasibility of integrating galactose-targeting strategies with photodynamic therapy. Therefore, coupling the galactose-targeting unit with a photosensitizer, photothermal agent, or prodrug to develop a diagnostic-therapeutic probe capable of both imaging and treatment may become a reality, thereby enabling simultaneous imaging and treatment of HCC.

**TABLE 2:** Summary of the photophysical properties and biological validation levels of representative probes.

Probe name	λ_ex_ (nm)	λ_em_ (nm)	Limit of detection	Response time	Biological validation level	References
DCM-Mor-Gal	560	690	7.60 × 10^−3^ U/mL	15 min	*in vitro,* *in vivo*	Yi et al. (2025)
LDMTY	535	561	2.54 × 10^−4^ U/mL	30 min	*in vitro,* *in vivo*	Liao et al. (2025)
TCF-Gal-Cys	576	645	0.80 × 10^−7^ M	10 min	*in vitro,* *in vivo*	Linghu et al. (2024)
DCM-Cys-Gal	558	723	7.70 × 10^−7^ M	20 min	*in vitro,* *in vivo*	Li et al. (2024b)
QL-Gal-N_3_	365	521	1.26 × 10^−7^ M	1.0 min	*in vitro*	Shen et al. (2020)
MOR-HOCl-Gal	490	560	1.40 × 10^−7^ M	1.0 min	*in vitro,* *in vivo*	Qin et al. (2024)
TP-Gal-1	405	530	7.90 × 10^−8^ M	2.0 min	*in vitro,* *in vivo*	Liu et al. (2023)
TP-Gal-2	405	530	5.06 × 10^−8^ M	2.0 min	*in vitro,* *in vivo*	Liu et al. (2023)
TP-Gal-3	405	530	1.04 × 10^−7^ M	2.0 min	*in vitro,* *in vivo*	Liu et al. (2023)
Rho-Gal	564	582	9.49 × 10^−8^ M	_	*in vitro*	Hu and Wang (2022)
Rho-Lac	564	582	1.37 × 10^−8^ M	_	*in vitro*	Hu and Wang (2022)
Rho-Glu	564	582	1.33 × 10^−8^ M	_	*in vitro*	Hu and Wang (2022)
NFP-G	440	541	1.20 × 10^−7^ M	60 min	*in vitro*	Zhou et al. (2021)
HT-V	500	616	_	_	*in vitro,* *in vivo*	Li et al. (2022)

**TABLE 3:** Summary of the targeting validation methods and imaging models of representative probes.

Probe name	Targeting validation method	Imaging model	References
DCM-Mor-Gal	Cell selectivity (fluorescence signals): HepG2 > others; H22 mouse organ distribution (liver > others); zebrafish liver-specific accumulation	FLI	Yi et al. (2025)
LDMTY	Cell selectivity (fluorescence signals): HepG2 > others; mice organ distribution (liver > others)	FLI	Liao et al. (2025)
TCF-Gal-Cys	Cell selectivity (fluorescence signals): HepG2 > others; zebrafish liver-specific accumulation	FLI	Linghu et al. (2024)
DCM-Cys-Gal	Cell selectivity (fluorescence signals): HepG2 > others; zebrafish liver-specific accumulation	FLI	Li et al. (2024b)
QL-Gal-N_3_	Cell selectivity (fluorescence signals): HepG2 > others	FLI	Shen et al. (2020)
MOR-HOCl-Gal	Cell selectivity (fluorescence signals): HepG2 > others; zebrafish liver-specific accumulation	FLI	Qin et al. (2024)
TP-Gal-1	Cell selectivity (fluorescence signals): HepG2 > others; mice organ distribution (liver > others)	FLI	Liu et al. (2023)
TP-Gal-2	Same as TP-Gal-1 but fluorescence signals of TP-Gal-1 < TP-Gal-2	FLI	Liu et al. (2023)
TP-Gal-3	Same as TP-Gal-1 but fluorescence signals of TP-Gal-1 < TP-Gal-2 < TP-Gal-3	FLI	Liu et al. (2023)
Rho-Gal	Cell (HepG2) fluorescence signals: Rho-Gal > Rho-Lac > Rho-Glu	FLI	Hu and Wang (2022)
Rho-Lac	_	FLI	Hu and Wang (2022)
Rho-Glu	_	FLI	Hu and Wang (2022)
NFP-G	Cell selectivity (fluorescence signals): HepG2 > others; loss-of-function strategy using β-galactosidase to remove the galactose moiety	FLI	Zhou et al. (2021)
HT-V	Cell selectivity (fluorescence signals): HepG2 > others; D-galactose competitive inhibition	FLI	Li et al. (2022)

FLI, fluorescence imaging.

**TABLE 4:** Summary of the *in vivo* applicability and biological models of representative probes.

Probe name	*In vivo* applicability	Response mechanism	Biological model	Detected analyte	References
DCM-Mor-Gal	H22 mice (*ex vivo*), zebrafish	Intramolecular charge transfer (ICT)	HepG2/HL-7702/A549/HeLa/SGC7901, H22 mice, zebrafish	β-Gal	Yi et al. (2025)
LDMTY	HCC subcutaneous tumor-bearing nude mice; zebrafish	ICT	LO2, HepG2, HONE1 cells; HCC subcutaneous tumor-bearing nude mice; zebrafish	β-Gal and HClO	Liao et al. (2025)
TCF-Gal-Cys	Zebrafish	ICT	HepG2, A549, HeLa, SGC-7901 cells; zebrafish	Cys	Linghu et al. (2024)
DCM-Cys-Gal	Zebrafish	ICT	HepG2, A549, HeLa, SGC cells; zebrafish	Cys	Li et al. (2024b)
QL-Gal-N_3_	No	Photoinduced electron transfer (PET)	HepG2, A549, HeLa cells	H_2_S	Shen et al. (2020)
MOR-HOCl-Gal	Zebrafish	ICT	HepG2, A549, MCF7, LoVo cells; zebrafish	ClO^−^	Qin et al. (2024)
TP-Gal-1	Kunming mice	PET	HepG2, HeLa, A549, SGC7901 cells; Kunming mice	Fe^3+^	Liu et al. (2023)
TP-Gal-2	Kunming mice	PET	HepG2, HeLa, A549, SGC7901 cells; Kunming mice	Fe^3+^	Liu et al. (2023)
TP-Gal-3	Kunming mice	PET	HepG2, HeLa, A549, SGC7901 cells; Kunming mice	Fe^3+^	Liu et al. (2023)
Rho-Gal	No	PET	HepG2, HeLa, SGC7901 cells	Hg^2+^	Hu and Wang (2022)
Rho-Lac	No	PET	HepG2, HeLa, SGC7901 cells	Hg^2+^	Hu and Wang (2022)
Rho-Glu	No	PET	HepG2, HeLa, SGC7901 cells	Hg^2+^	Hu and Wang (2022)
NFP-G	No	PET	HepG2, HeLa, L929, A549 cells	Formaldehyde	Zhou et al. (2021)
HT-V	Nude mouse	Twisted ICT	HepG2, HL7702, HeLa, 4T-1, A549 cells; nude mouse	Viscosity	Li et al. (2022)

In summary, galactose-based small-molecule fluorescent probes that target hepatocytes show great promise for the early diagnosis of HCC. Through systematic progress in the various areas mentioned herein, this field is expected to achieve successful transition from the laboratory to clinical practice in the near future, providing strong technical support for the early diagnosis, efficacy assessment, and personalized treatment of HCC.
